# Activation of MEK1 or MEK2 isoform is sufficient to fully transform intestinal epithelial cells and induce the formation of metastatic tumors

**DOI:** 10.1186/1471-2407-8-337

**Published:** 2008-11-17

**Authors:** Laure Voisin, Catherine Julien, Stéphanie Duhamel, Kailesh Gopalbhai, Isabelle Claveau, Marc K Saba-El-Leil, Ian Gaël Rodrigue-Gervais, Louis Gaboury, Daniel Lamarre, Mark Basik, Sylvain Meloche

**Affiliations:** 1 Institut de Recherche en Immunologie et Cancérologie, Montreal, Quebec, Canada; 2 Department of Molecular Biology, Université de Montréal, Montreal, Quebec, Canada; 3 Department of Pharmacology, Université de Montréal, Montreal, Quebec, Canada; 4 Laboratoire d'Immunovirologie, Centre de Recherche du CHUM, Université de Montréal, Montreal, Quebec, Canada; 5 Jewish General Hospital, McGill University, Montreal, Quebec, Canada

## Abstract

**Background:**

The Ras-dependent ERK1/2 MAP kinase signaling pathway plays a central role in cell proliferation control and is frequently activated in human colorectal cancer. Small-molecule inhibitors of MEK1/MEK2 are therefore viewed as attractive drug candidates for the targeted therapy of this malignancy. However, the exact contribution of MEK1 and MEK2 to the pathogenesis of colorectal cancer remains to be established.

**Methods:**

Wild type and constitutively active forms of MEK1 and MEK2 were ectopically expressed by retroviral gene transfer in the normal intestinal epithelial cell line IEC-6. We studied the impact of MEK1 and MEK2 activation on cellular morphology, cell proliferation, survival, migration, invasiveness, and tumorigenesis in mice. RNA interference was used to test the requirement for MEK1 and MEK2 function in maintaining the proliferation of human colorectal cancer cells.

**Results:**

We found that expression of activated MEK1 or MEK2 is sufficient to morphologically transform intestinal epithelial cells, dysregulate cell proliferation and induce the formation of high-grade adenocarcinomas after orthotopic transplantation in mice. A large proportion of these intestinal tumors metastasize to the liver and lung. Mechanistically, activation of MEK1 or MEK2 up-regulates the expression of matrix metalloproteinases, promotes invasiveness and protects cells from undergoing anoikis. Importantly, we show that silencing of MEK2 expression completely suppresses the proliferation of human colon carcinoma cell lines, whereas inactivation of MEK1 has a much weaker effect.

**Conclusion:**

MEK1 and MEK2 isoforms have similar transforming properties and are able to induce the formation of metastatic intestinal tumors in mice. Our results suggest that MEK2 plays a more important role than MEK1 in sustaining the proliferation of human colorectal cancer cells.

## Background

Colorectal cancer arises from intestinal epithelial cells in a multistep process that extend over several years and leads to the progression from a normal mucosa to aberrant crypt foci to benign adenomas up to invasive carcinomas [1]. Histo-pathological progression of colorectal tumors is associated with the progressive accumulation of genetic alterations in tumor suppressor genes and oncogenes [2]. The most frequently mutated oncogene in colorectal tumors is *KRAS*, a member of the *RAS *gene family. Activating mutations in the three *RAS *genes, most frequently in *KRAS*, have been found in ~30% of human neoplasias and are often an early event in tumor progression [3]. Specifically, *KRAS *mutations are detected in approximately 35% of all sporadic colorectal adenomas and carcinomas [3,4]. Genetic and biochemical studies have firmly established the central role of Ras GTPases in regulating cell proliferation, growth and survival [5,6]. More than ten distinct classes of Ras effectors have been identified to date, several of which are associated with oncogenic signaling pathways [7]. The best-characterized of the Ras effector pathways is the activation of the Raf family Ser/Thr kinases, leading to sequential phosphorylation and activation of MEK1/MEK2 and the mitogen-activated protein (MAP) kinases ERK1/ERK2 [8]. The importance of Raf in oncogenic signaling has been validated by the discovery of activating *BRAF *mutations in a variety of human tumors [9], including 14% of colorectal cancers [3].

Raf relays its oncogenic signals mainly via the MAP kinase kinases MEK1 and MEK2. Early studies have shown that expression of activated alleles of MEK1 is sufficient to deregulate the proliferation and trigger the morphological transformation of immortalized fibroblast [10,11] and epithelial [12-14] cell lines. *In vivo*, orthotopic transplantation of mammary epithelial cells expressing activated MEK1 into syngeneic mice rapidly produced invasive adenocarcinomas [13]. Transgenic expression of active MEK1 in mouse skin induced hyperplasia, hyperkeratosis and perturbed differentiation of the epidermis [15,16]. Conversely, treatment with MEK1/2 inhibitors was shown to inhibit the proliferation of various carcinoma and leukemic cell lines [17,18]. Notably, administration of an orally-available inhibitor of MEK1/2 elicited marked anti-tumor efficacy in mouse xenograft models of colon cancer and metastatic melanoma [19,20]. In parallel, several studies using clinical specimens have documented the up-regulation and/or activation of MEK1/MEK2 and the MAP kinases ERK1/ERK2 in solid tumors and leukemias (see [21] and references therein). Collectively, these findings have provided strong rationale for the development of small-molecule inhibitors of MEK1/2 for chemotherapeutic intervention in cancer.

MEK1 and MEK2 display 85% amino acid identity overall and are expressed ubiquitously in cell lines and tissues. Although it is commonly assumed that the two isoforms are functionally equivalent, several lines of evidence, however, indicate that they are regulated differentially and may exert non-redundant functions [22-24]. Studies using RNA interference have suggested that both MEK1 and MEK2 are required for *in vitro *cell proliferation, and that they contribute to distinct cell cycle regulatory events [25]. However, the individual roles of MEK1 and MEK2 in tumorigenesis remain to be explored. Intriguingly, a recent report has shown that activated MEK1 but not MEK2 can promote epidermal hyperplasia in transgenic mice, even though both MEK proteins trigger ERK1/ERK2 phosphorylation [16].

Colorectal cancers often display activation of the ERK1/2 MAP kinase pathway and therefore represent potential targets for MEK1/2 inhibitors [26]. In this study, we have evaluated the ability of the two MEK isoforms to transform intestinal epithelial cells and to promote tumor formation and progression *in vivo*. Our results demonstrate that activation of either MEK1 or MEK2 is sufficient for full transformation of intestinal epithelial cells up to the invasive stage. Importantly, we show that MEK2 expression is essential for the proliferation of human colon cancer cells.

## Methods

### Cell culture and infections

IEC-6 is a rat epithelial cell line with features of undifferentiated small intestinal crypt cells [27]. HCT116 [28], HT-29 [29] and SW480 [30] are human colorectal adenocarcinoma cell lines. MDA-MB-231 is a human breast adenocarcinoma cell line [31]. IEC-6, HCT116, HT-29 and MDA-MB-231 cells were cultured in DMEM containing 10% fetal bovine serum, 2 mM glutamine and antibiotics. SW480 cells were cultured in RPMI supplemented with 10% fetal bovine serum. IEC-6 cells were infected with retroviral vectors as previously described [32]. Populations of infected cells were selected with 4 μg/ml puromycine.

### Mouse *in vivo *tumor studies

All animals were housed under pathogen-free conditions, and experiments were performed in accordance with CCAC guidelines and with Université de Montréal Institutional Animal Care and Use Committee approval. Female Balb/c athymic nude mice (nu/nu) were purchased from Harlan and used at 6–8 weeks of age. For subcutaneous tumor model studies, IEC-6 cells were harvested from sub-confluent cultures by brief exposure to 0.25% trypsin and 0.02% EDTA. The cells were washed once in PBS, and 3 × 10^4 ^cells in a final volume of 200 μl were injected subcutaneously in the flanks of the mouse. The mice were monitored regularly and the tumors were measured every 2–3 days using a caliper.

For orthotopic tumor model studies, 1 × 10^5 ^IEC-6 cells in a volume of 30 μl were implanted in the ceacum of nude mice. Mice were anaesthetized with isoflurane during the surgical procedure. The caecum was exteriorized through a small midline laparotomy and cells were injected in the cecal wall. After implantation, the abdominal wall was closed and sutured, and the mice received a subcutaneous injection of 0.05 mg/kg buprenorphin for postoperative pain relief. The mice were monitored regularly and sacrificed when they became moribund or manifested signs of disease. Following necropsy, the caecum, lungs and liver were excised, fixed in formalin and embedded in paraffin. Serial sections of the intestine and of lung and liver (0.3 mm apart) tissues were prepared and stained by H&E for histopathological evaluation.

### Plasmids and antibodies

Human HA-tagged MEK1 and MEK2 cDNA constructs were used as templates for *in vitro *mutagenesis to generate the constitutively activated MEK1(S218D/S222D) (MEK1DD) and MEK2(S222D/S226D) (MEK2DD) mutants as previously reported [33]. All mutations were confirmed by DNA sequencing. All MEK constructs were subcloned into pBabe-puro vector for infection of IEC-6 cells.

Commercial antibodies were from the following sources: Bcl-2, Mcl-1 and GAPDH (FL-335) antibodies from Santa-Cruz Biotechnology; smooth muscle α-actin (clone 1A4), total actin (clone AC40) and α-tubulin (clone DM1A) from Sigma ; MEK1 (clone 25), MEK2 (clone 96), fibronectin (Clone 10) and E-cadherin (clone 36 for immunofluorescence and clone 34 for immunoblotting) from Transduction Laboratories; pan-cytokeratin (clone B311.1) and MMP-13 from Calbiochem ; vimentin from Chemicon (for immunofluorescence) and NeoMarkers (Ab-2; for immunoblotting); phospho-MEK1/2, Bcl-xL and Bim from Cell Signaling Technology.

### Immunoblotting, protein kinase assays and immunofluorescence analysis

Cell lysis, immunoprecipitation and immunoblot analysis were performed as described previously [34]. The phosphotransferase activity of ectopically expressed MEK1 and MEK2 was assayed by measuring their ability to increase the myelin basic protein kinase activity of recombinant ERK2 *in vitro *as previously described [35].

Immunofluorescence staining was performed as described [36]. Cell samples were viewed by fluorescence microscopy on a Leica DM IRB microscope.

### Real-time quantitative PCR analysis

Total RNA was isolated using the RNeasy Mini Kit (Qiagen) and was reversed transcribed and amplified using primers-probe set from Exiqon Universal ProbeLibrary. Real-time analysis of PCR product amplification was performed on the ABI PRISM 7900HT Sequence Detection System (Applied Biosystems). The mouse ribosomal 18S gene was used as endogenous control. The relative level of target gene expression was quantified using the ΔΔCT method.

### Cell proliferation and transformation assays

Cell proliferation *in vitro *was measured by the colorimetric MTT assay. Briefly, exponentially growing cells were cultured in 24-well plates in complete DMEM medium. Cell proliferation was determined at 24 h intervals by replacing the culture medium with 0.05 ml of MTT solution (1 mg/ml MTT). The cells were then incubated at 37°C for 1 h prior to addition of 100 μl of the solubilizer solution (DMSO with 2% (v/v) of glycine 0.1 M, pH 11). The absorbance was determined at 550 nm with reference at 620 nm.

Anchorage independence growth was evaluated as originally described [37]. Briefly, cells (2.5 × 10^4^) were resuspended in 2 ml of top agar (0.4% Noble agar (Difco) in DMEM, 10% calf serum, 2 mM glutamine, and antibiotics) and overlaid on a solid layer of 5 ml of 0.7% agar in the same medium in 60-mm tissue culture plates. The cells were fed weekly with 1 ml of top agar in complete medium. The plates were examined for the presence of colonies after 21 days.

### Migration and invasion assays

Cell migration and invasion were determined using a modified two-chamber migration assay. Membranes (8-μm pore size; Neuro Probe) were either coated with 50 mg/ml collagen type I (migration assays) or with a layer of Matrigel extracellular matrix proteins (BD Bioscience) for invasion assays. The cells (5.5 × 10^4^) were seeded in serum-free medium in the upper chamber and allowed to migrate to the lower chamber containing 10% fetal bovine serum as chemoattractant. After 6 h, the cells in the upper chamber were carefully removed using a wiper blade and the cells on the bottom side of the membrane were fixed and stained with Diff-Quick Stain Set (Fisher). The stained membrane was then digitally scanned and the density of cells was quantified using the NIH Image software. Essentially similar results were obtained when the stained cells were counted manually.

### Detachment-induced apoptosis assay

Tissue culture plates were coated twice with 5 mg/ml Poly-HEMA (Sigma), allowed to dry at room temperature and rinsed with PBS. IEC-6 cells were added to the coated plates in complete growth medium at a density of 4 × 10^4 ^cells/cm^2 ^for the indicated times. The cells were then harvested, rinsed with PBS and counted. Apoptosis was measured on an aliquot of 10^4 ^cells using a cell death detection ELISA kit (Roche Diagnostics) according to the manufacturer's procedure.

### Gelatin and casein zymography assays of MMPs

Conditioned medium harvested from IEC-6 cells was mixed with 2× Laemmli's sample buffer and incubated on ice for 10 min. The samples were analyzed by electrophoresis on a 10% SDS-acrylamide gel containing 1 mg/ml gelatin (Sigma) or on a Novex 12% Zymogram (casein) Gel (Invitrogen). The gel was washed twice in 2.5% Triton X-100 for 30 min to remove all traces of SDS, and then incubated overnight at 37°C in 50 mM Tris-HCl (pH 7.6), 10 mM CaCl_2_, 100 mM NaCl and 0.05% Brij35. Then, the gel was stained with 0.05% Coomassie, destained and dried.

### shRNA lentiviral infections

The shRNA constructs for human MEK1 (*MAP2K1*) and MEK2 (*MAP2K2*) genes were purchased from Open Biosystems. These constructs from The RNAi Consortium (TRC) library consist of 21 base stem and 6 base loop hairpins cloned in pLKO1 lentiviral vector. The HIV packaging (pMDLg/p REE and pRSV-REV) and VSV-G (pMD2-VSVG) plasmids were kindly provided by D. Trono (École Polytechnique Fédérale de Lausanne). For lentivirus production, 2 × 10^6 ^293T cells were cultured overnight in T25 flasks and co-transfected with 6 μg of plasmid vector, 1.5 μg of pMDLg/p REE, 3 μg of pMD2-VSVG and 1.5 μg of pRSV-REV using the calcium phosphate precipitation method. Viral supernatants were collected after 42 h. For infection of human carcinoma cell lines, cells were plated at a density of 1–4.5 × 10^6 ^cells per 10-cm Petri dish the day before, and were then incubated with viral supernatant in the presence of 4 μg/ml polybrene for 5 h. After infection, the cells were washed twice with PBS, and cultured for 72 h before harvesting. The yield of infection was estimated by parallel infection of the cells with a GFP-encoding lentiviral vector. A similar efficiency of transduction was obtained in the colorectal and breast cancer cell lines used in this study.

### Affymetrix GeneChip analysis

Total RNA was isolated from empty vector-, MEK1DD-, and MEK2DD-expressing IEC-6 cells (duplicate samples) using RNeasy RNA isolation kit (Qiagen). The quality of the RNA was assessed by determining the 260/280 nm absorbance ratios and by gel electrophoresis in agarose/formaldehyde gels. Reverse transcription, second-strand synthesis, and cRNA labeling were all performed using standard Affymetrix protocols. Biotinylated cRNAs were hybridized to rat Genome U34A GeneChips (Affymetrix) on an Affymetrix Fluidics Station at the McGill Genome Centre. After scanning of the gene chips, images were analyzed and the expression values were normalized using the Affymetrix Microarray Analysis Suite (v5.0). The resulting expression values were analyzed using empirical Bayes methodology [38].

## Results

### Constitutive activation of MEK1 or MEK2 is sufficient for transformation of intestinal epithelial cells and formation of tumors *in vivo*

**Figure 1:**
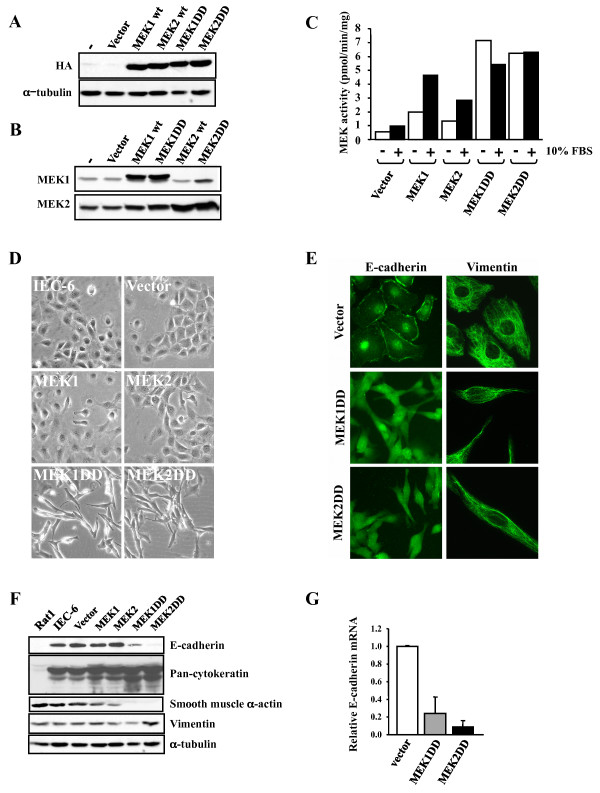
**Expression of activated MEK1 or MEK2 morphologically transforms intestinal epithelial cells**. IEC-6 cells were infected with retroviruses encoding the indicated MEK constructs and populations of puromycine-resistant cells were selected for further analysis. (A) Expression of HA-tagged MEK1 and MEK2 was analyzed by immunoblotting with anti-HA antibody. (B) Expression of MEK1 and MEK2 was analyzed by immunoblotting with specific antibodies. (C) The ectopically expressed MEK proteins were immunoprecipitated with anti-HA antibody, and phosphotransferase activity was measured using an ERK2 reactivation assay. Results are representative of at least three independent experiments. (D) Morphology of exponentially proliferating IEC-6 cells stably expressing the indicated MEK constructs was examined by phase-contrast microscopy. (E) Immunofluorescence analysis of E-cadherin and vimentin expression. (F) Expression of E-cadherin, cytokeratins, vimentin and smooth muscle α-actin was analyzed by immunoblotting in the indicated cell lines. α-Tubulin was used as loading control. (G) Quantitative PCR analysis of E-cadherin mRNA levels. Expression levels are expressed as fold-increase relative to vector-infected cells.

Immunohistochemistry analysis of a colorectal cancer tissue microarray containing over 400 colorectal cancer and 50 normal colon tissue biopsies revealed that 44% of colorectal cancers display high cytoplasmic expression of phosphorylated MEK1/MEK2 as compared to 10% of normal tissues (analysis to be published elsewhere). To assess the functional significance of MEK1/MEK2 activation in colorectal cancer, we ectopically expressed wild type and constitutively active (DD mutant) versions of MEK1 and MEK2 by retroviral gene transfer in the normal undifferentiated intestinal epithelial cell line IEC-6 [27]. Polyclonal populations of infected clones were selected in puromycin and used for subsequent experiments. Immunoblot analysis confirmed that ectopic MEK isoforms are expressed at comparable levels in IEC-6 transduced populations (Fig. 1A). Overexpression of wild type MEK1 or MEK2 did not affect the expression of endogenous MEK isoforms. However, ectopic expression or MEK2DD slightly increased the steady-state levels of endogenous MEK1, while overexpression of MEK1DD had a similar effect on MEK2 levels (Fig. 1B). As expected, substitution of the activation loop Ser phosphorylation sites by Asp residues strongly potentiated the enzymatic activity of MEK1 and MEK2, but no reproducible difference in activity was observed between the two isoforms (Fig. 1C).

IEC-6 cells grow as a monolayer and display a typical epithelial morphology with organized cell-cell adhesions (Fig. 1D). Overexpression of wild type MEK isoforms had no noticeable effect on the morphology of IEC-6 cells. In contrast, expression of activated MEK1 or MEK2 led to drastic morphological changes accompanied by loss of cell-cell contacts; the cells adopted a spindle-like fibroblast morphology, were more refractile and formed multilayers. These changes are characteristic of epithelial-mesenchymal transitions (EMT) that play an important role in tumor progression [39]. To determine whether MEK1DD- and MEK2DD-expressing cells undergo an EMT, we examined the localization and measured the expression levels of various epithelial and mesenchymal markers. Parental and vector-infected IEC-6 cells showed a polarized basolateral membrane distribution of the epithelial marker E-cadherin, with basal expression of the fibroblast marker vimentin (Fig. 1E). Ectopic expression of MEK1DD or MEK2DD resulted in the loss of E-cadherin staining at the plasma membrane (Fig. 1E), concomitant with a marked reduction of E-cadherin protein and mRNA levels (Fig. 1F and 1G). No significant change in the expression of keratins and no induction of the mesenchymal proteins vimentin and smooth muscle α-actin (we instead observed a decreased expression) were observed in these cells (Fig. 1F). These results indicate that constitutive activation of MEK1 or MEK2, while disrupting normal epithelial morphology and polarization, is not sufficient to induce a full EMT in intestinal epithelial cells. This epithelial plasticity change has been referred to as scattering and is distinct from EMT [40].

**Figure 2:**
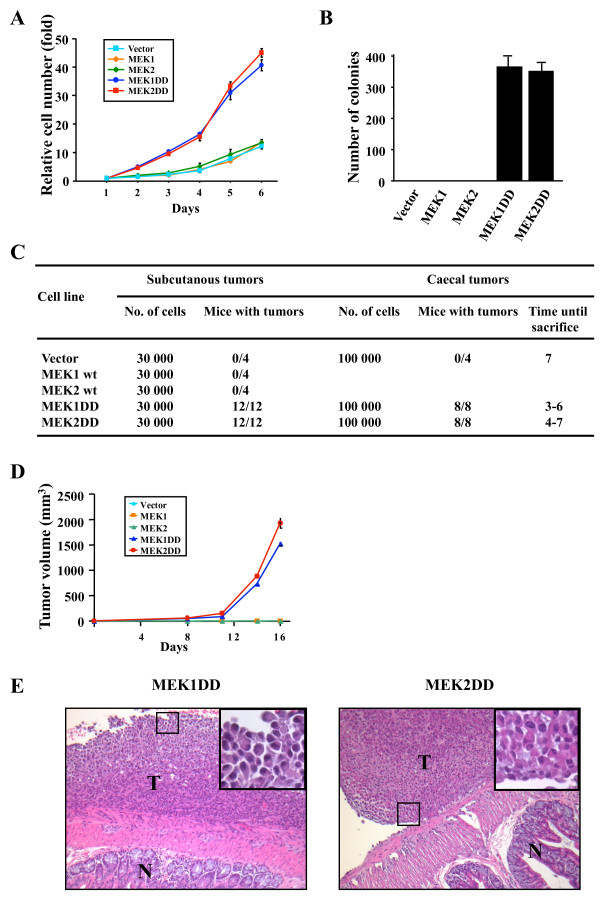
**Expression of activated MEK1 or MEK2 enhances the proliferation of IEC-6 cells and induces the formation of intestinal tumors**. (A) IEC-6 cells infected with the indicated constructs were plated in triplicate wells of 24-well plates in complete medium containing 10% serum. Cell proliferation was measured each day using the MTT assay. (B) Anchorage-independent proliferation was assayed by measuring the formation of cell colonies in soft agar. Results indicate the number of colonies after 21 days and are representative of 3 independent experiments. (C) IEC-6 cell populations expressing the indicated MEK constructs were either injected subcutaneously (subcutaneous tumors) into the flanks of athymic mice or orthotopically transplanted into the cecal wall (cecal tumors) of the mice as described in Methods. The mice were monitored for tumor development. (D) Growth rate of subcutaneous tumors. Values represent the average volume +/- SEM of 8 tumors (4 mice). Data are representative of 3 independent experiments. (E) Representative pictures of intestinal tumors generated by orthotopic implantation of MEK1DD- or MEK2DD-expressing IEC-6 cells in the ceacum. N, normal mucosa; T, tumor. Note the poorly differentiated morphology of tumor cells typical of high-grade adenocarcinomas. Inset, signet-ring cells.

We examined whether constitutive activation of MEK1 or MEK2 was conferring some proliferation advantage to intestinal epithelial cells. Ectopic expression of either activated MEK1 or MEK2 significantly increased the proliferation rate of IEC-6 cells grown in 10% serum containing-medium when compared to vector-infected cells or cells overexpressing wild type MEK isoforms (Fig. 2A). This increase in proliferation was not observed in low serum (2%) containing-medium (data not shown). Both activated MEK1 and MEK2 conferred anchorage-independence growth to IEC-6 cells (Fig. 2B). To test the tumorigenic potential of IEC-6 transduced cell populations *in vivo*, the cells were injected subcutaneously into athymic mice. Cells infected with vector or wild type MEK isoforms never formed any tumor (Fig. 2C). In contrast, both MEK1DD- and MEK2DD-expressing cells generated rapidly growing tumors in all injected mice. Injection of as low as 3 × 10^4 ^cells produced tumors of ~1,000 mm^3 ^after 2 weeks. No major difference was observed in the growth rate of tumors expressing activated MEK1 or MEK2 (Fig. 2D).

To analyze the impact of active MEK isoforms on tumorigenesis in a more pathologically relevant model, IEC-6 transduced cells were orthotopically transplanted into the caecum of athymic mice. This model more closely recapitulates human colorectal cancer progression, in particular the spontaneous metastatic process that is highly dependent on the host environment [41]. Strikingly, 100% of the mice transplanted with 10^5 ^IEC-6 cells expressing either MEK1DD or MEK2DD developed massive intestinal tumors, while the control group remained tumor-free (Fig. 2C and 2E). The mice were sacrificed when they became moribund or presented symptoms of weight loss, respiratory distress, or a palpable abdominal mass. Microscopic examination of the tumors revealed a poorly differentiated morphology with occasional signet-ring cells (inset) corresponding to a high-grade adenocarcinoma (Fig. 2E). The tumors are invasive and diffusely infiltrate the lamina propria and the underlying muscular layers (muscularis propria and muscularis mucosae). Together, these data demonstrate that constitutive activation of MEK1 or MEK2 is sufficient to transform intestinal epithelial cells and induce the formation of invasive colon adenocarcinomas.

### Constitutive activation of MEK1 or MEK2 confers metastatic properties to transformed intestinal epithelial cells

**Figure 3:**
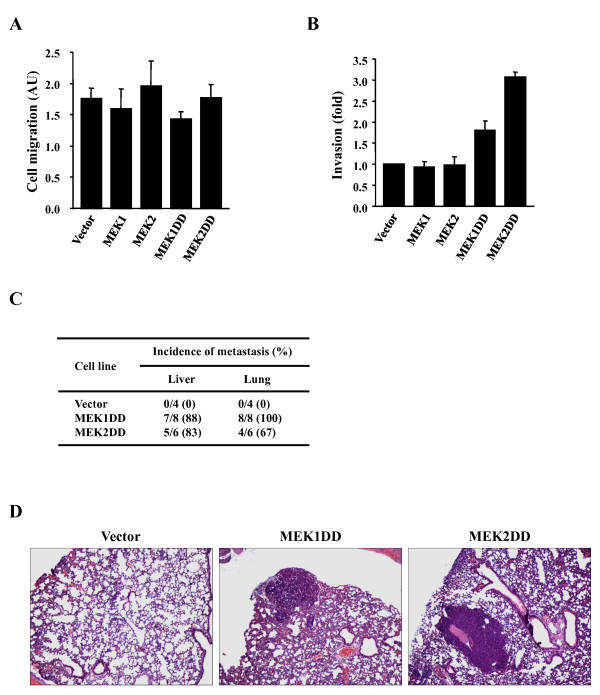
**Constitutive activation of MEK1 or MEK2 promotes tumor metastasis to the liver and lung**. (A) Motility of IEC-6 cell populations was assayed by measuring their migration through porous membranes coated with collagen type I in modified Boyden chambers. Cells on the bottom side of the membrane were fixed and stained. (B) Invasiveness of IEC-6 cell populations was assayed as in A except that membranes were coated with a layer of Matrigel extracellular matrix proteins. Results are expressed as fold-increase relative to vector-infected cells. (C) Incidence of metastasis following orthotopic transplantation of IEC-6 cell populations in the caecum of mice. (D) Representative pictures of lung metastases derived from intestinal tumors expressing activated MEK1 or MEK2.

Activation of the ERK1/2 MAP kinase pathway has been implicated in the regulation of cell motility and invasion [42]. Notably, treatment of colon carcinoma cells with the MEK1/2 inhibitor PD184352 was shown to inhibit HGF-induced cell scattering and to reduce their invasive properties [20]. We examined the impact of MEK1 or MEK2 activation on the motility of IEC-6 cells using two different cell migration assays. No difference in the migration rate of the different IEC-6 transduced populations was observed in a standard chemotaxis assay with serum as chemoattractant (Fig. 3A). Similar results were obtained using a wound-healing assay (data not shown). We next analyzed the ability of the cells to migrate through a Matrigel-coated membrane as a reflection of their invasive properties. Ectopic expression of activated MEK1 or MEK2 significantly enhanced the invasive capacity of IEC-6 cells, while the wild type MEK isoforms had no effect (Fig. 3B). Interestingly, the MEK2DD-transduced cells appeared more invasive than cells expressing MEK1DD in this assay.

The invasive properties of the cells *in vitro *and the histology of the intestinal tumors suggest that MEK1DD- and MEK2DD-expressing IEC-6 cells may have metastatic properties *in vivo*. Detailed histological examination of a subset of mice that develop orthotopic tumors revealed the presence of metastasis in the lymph nodes, the lungs and the liver in both the MEK1DD and MEK2DD groups (Fig. 3C and 3D). These observations indicate that constitutive activation of either MEK1 or MEK2 is sufficient to confer a metastatic phenotype to intestinal tumor cells. The acquisition of invasiveness does not result from changes in cellular motility.

**Figure 4:**
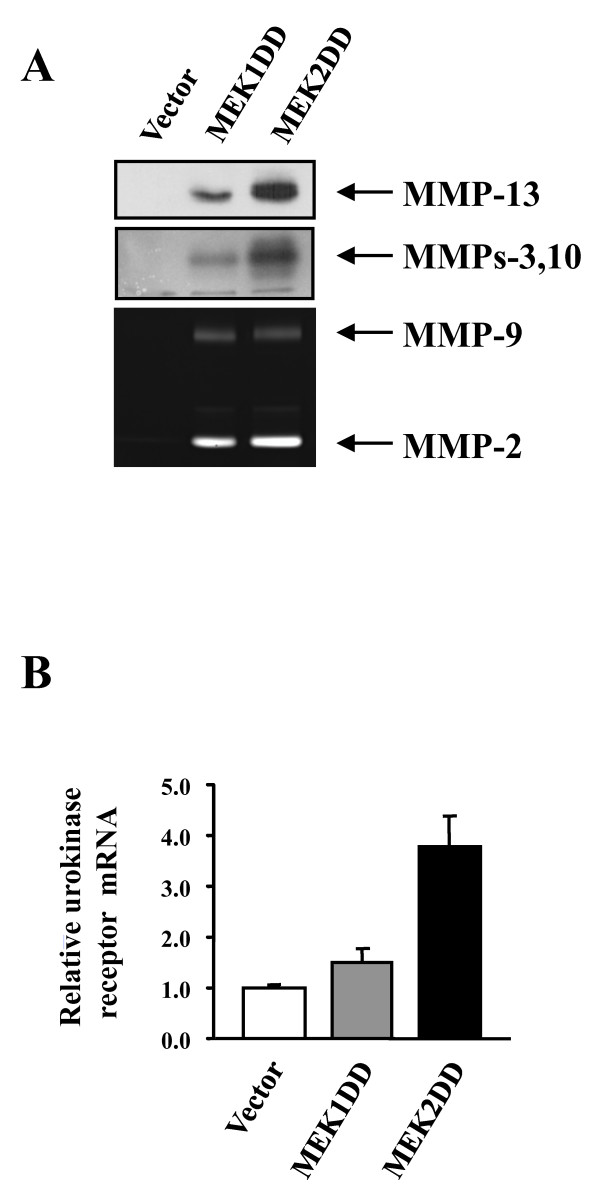
**Expression of proteases in IEC-6 cell populations transduced with activated MEK isoforms**. (A) Expression of MMP-13 was analyzed by immunoblotting. Induction of MMP activity was analyzed by zymography on acrylamide gels co-polymerized with casein (MMP-3/10) or gelatin (MMP2 and MMP9) substrates. Photographs of the gels are shown. (B) Quantitative PCR analysis of urokinase receptor mRNA levels. Expression levels are expressed as fold-increase relative to vector-infected cells.

To identify downstream targets of MEK1/MEK2 involved in intestinal tumor progression, we analyzed the transcriptional profile of MEK1DD- and MEK2DD-expressing IEC-6 cells using Affymetrix GeneChip arrays. Analysis of the gene expression data identified several genes that were up-regulated or down-regulated in MEK1DD- and MEK2DD-expressing cells as compared to control IEC-6 cells (Additional files 1 and 2). The list of modulated genes included growth factors, signaling molecules, drug metabolism enzymes and, interestingly, several proteases. The matrix metalloproteinases (MMPs) MMP-3 and MMP-13 were up-regulated in both MEK1DD- and MEK2DD-expressing cells, while up-regulation of MMP-10 reached significance only in MEK2DD cells. Expression of the urokinase receptor was also up-regulated in IEC-6 cells expressing activated MEK2. Because of the importance of MMPs and urokinase receptor in tumor progression [43,44], we further validated the regulation of these genes by MEK1 and MEK2 signaling to confirm the data from the arrays. No expression or activity of MMPs could be detected in empty vector-infected IEC-6 cells. However, activation of either MEK1 or MEK2 markedly up-regulated the expression of MMP-13 protein (Fig. 4A). Notably, higher levels of MMP-13 protein were detected in IEC-6 cells expressing the activated MEK2 isoform. The expression of MMP-3/10 was analyzed by measuring their activity by zymography in casein-containing gels. Again, we observed that MEK2DD increased MMP-3/10 enzymatic activity more robustly than MEK1DD (Fig. 4A). Quantitative PCR analysis confirmed that constitutive activation of MEK1 or MEK2 induces the expression of urokinase receptor mRNA (Fig. 4B). As observed for the MMPs, the extent of induction of the urokinase receptor gene was higher in MEK2DD-expressing cells.

In a previous study, Komatsu et al. [45] have used oligonucleotide microarrays to analyze the gene expression profile of intestinal epithelial cells expressing a conditional allele of activated MEK1. We have compared the results of our transcriptional profiling analysis with this study. Of the 69 gene transcripts that showed altered expression in the study of Komatsu, 18 (26%) were found to be modulated in IEC-6 cells expressing constitutively active MEK1 or MEK2. Importantly, the two studies converge on a series of genes involved in cell proliferation, cell invasion, tumor suppression and drug metabolism.

### Constitutive activation of MEK1 or MEK2 protects intestinal epithelial cells against anoikis

**Figure 5:**
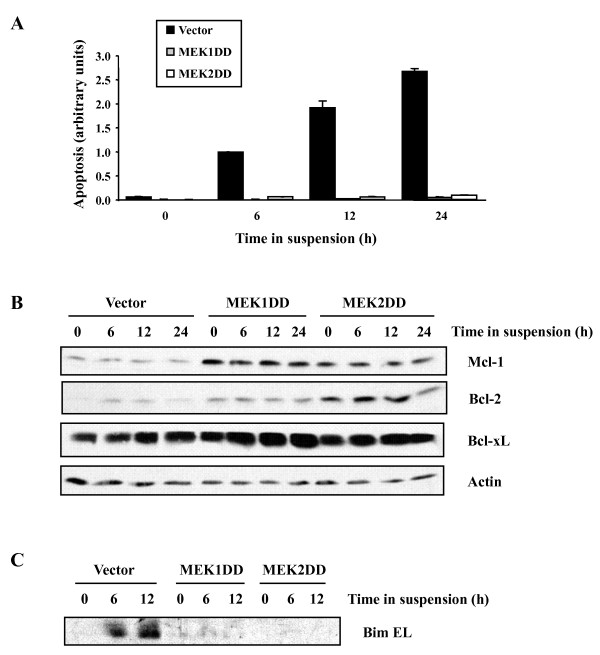
**Constitutive activation of MEK1 or MEK2 confers intestinal epithelial cells resistance to anoikis**. (A) IEC-6 cell populations infected with vector alone or activated MEK1 or MEK2 were cultured in suspension on poly-HEMA-coated plates for the indicated times. Apoptosis was evaluated by TUNEL assay. (B and C) Expression of Bcl-2 anti-apoptotic (B) and pro-apoptotic (C) family proteins was analyzed by immunoblotting in cell extracts from IEC-6 cell populations described in A. Total actin was used as loading control.

Epithelial cancer progression and metastasis is associated with the acquisition of resistance to anoikis [46]. To further explore the mechanism by which MEK1 and MEK2 promote tumor metastasis, we asked whether activated MEK isoforms protect intestinal epithelial cells from cell death induced by loss of adhesion. IEC-6-transduced populations were placed on poly-HEMA-coated plates in normal growth medium and the extent of apoptosis was measured at different times by TUNEL. Detachment from matrix induced high levels of apoptosis of control IEC-6 cells, which was already detectable at 6 h and increased up to 24 h (Fig. 5A). Strikingly, expression of either MEK1DD or MEK2DD almost completely protected IEC-6 cells from undergoing anoikis.

As a step to understand the molecular mechanism by which activated MEK isoforms suppress anoikis, we monitored the expression of Bcl-2 anti-apoptotic and pro-apoptotic family proteins. Constitutive activation of MEK1 or MEK2 resulted in the up-regulation of the pro-survival proteins Mcl-1, Bcl-2 and, to a lesser extent, Bcl-x_L _in IEC-6 cells (Fig. 5B). Our results confirm and extend previous observations [47,48] by demonstrating that both MEK1 and MEK2 isoforms share the property to induce the accumulation of Bcl-2 family pro-survival members. Reciprocally, induction of the BH3-only pro-apoptotic protein Bim was completely suppressed in cells expressing MEK1DD or MEK2DD (Fig. 5C). This finding is consistent with previous reports documenting the role of the ERK1/2 MAP kinase pathway in promoting the degradation of Bim [49,50]. MEK1 or MEK2 activation had no significant effect on the expression of the pro-apoptotic proteins Bax and Bak in these cells (data not shown).

### Silencing of MEK2 expression markedly inhibits the proliferation of human colon cancer cells

**Figure 6:**
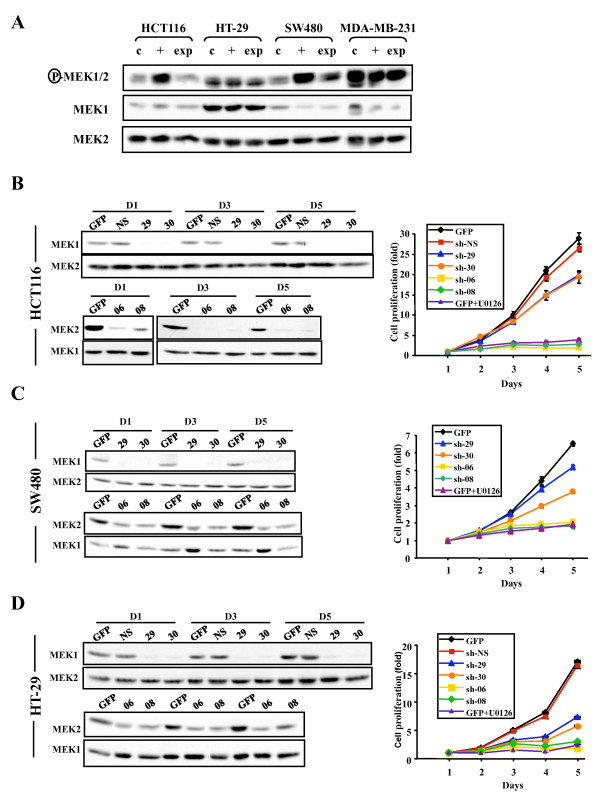
**Impact of MEK1 or MEK2 silencing on the proliferation of human colon carcinoma cell lines**. (A) Immunoblot analysis of MEK1, MEK2 and phospho-MEK1/2 expression in the human colon carcinoma cell lines HCT116, HT-29 and SW480, and in the breast adenocarcinoma line MDA-MB-231. Lysates were prepared from cells deprived of serum for 24 h (c), restimulated with serum for 5 min (+), or proliferating exponentially (exp). (B to D) HCT116 (B), SW480 (C) and HT-29 (D) cells were infected with MEK1 or MEK2 shRNA-encoding lentiviruses. A non-silencing inactive MEK1 shRNA (NS) was used as negative control. The cells were replated 72 h (day 0) after infection to monitor the expression of MEK1 and MEK2 and to measure the rate of cell proliferation. Expression of MEK isoforms was analyzed by immunoblotting at day (D) 1, 3 and 5 (left). Cell proliferation was measured each day using the MTT assay (right). For experiments with small-molecule MEK1/2 inhibitor, the cells were treated with 10 μM U0126 added at 48 h intervals.

**Figure 7:**
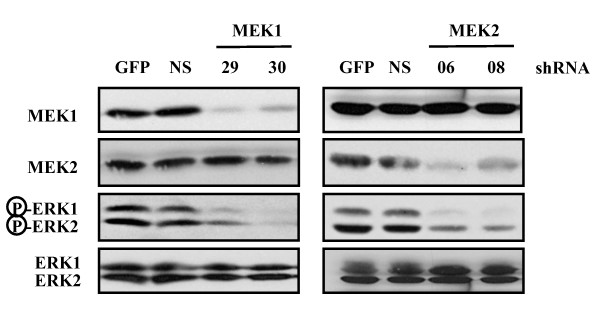
**Silencing of MEK1 or MEK2 expression restrains activating phosphorylation of the MAP kinases ERK1/ERK2**. HCT116 cells were infected with lentiviruses encoding shRNAs to MEK1 or MEK2 gene. The expression of MEK1/MEK2, ERK1/ERK2 and phospho-ERK1/2 was measured by immunoblotting at day 1 as described in the legend of Figure 6. NS, non-silencing inactive MEK1 shRNA.

The results presented above clearly demonstrate that constitutive activation of either MEK isoform, MEK1 or MEK2, is sufficient to fully transform intestinal epithelial cells to the metastatic stage. We next wanted to determine if human colon cancer cells depend on the activity of MEK isoforms for cell proliferation. Several human colon carcinoma cell lines display constitutive phosphorylation of ERK1/ERK2 MAP kinases [20], likely resulting from activation of MEK1/MEK2. The HCT116 cell line, which represents one of the best studied model of colorectal cancer cells, display constitutive activation of the two MEK isoforms (Fig. 6A). To assess the individual roles of MEK1 and MEK2, we expressed short-hairpin (sh) RNAs specifically targeting MEK1 or MEK2 gene in HCT116 cells using VSV-pseudotyped lentiviral vectors. We tested the effect of 5 distinct shRNAs for MEK1 and 3 shRNAs for MEK2, using as control a GFP-encoding vector. We selected the two most efficient shRNAs to MEK1 and MEK2 genes (Additional file 3). A non-silencing inactive MEK1 shRNA was used as additional negative control in these experiments. The efficiency of transduction estimated by GFP immunofluorescence was over 90%, and therefore the experiments were performed without cellular selection. As shown by immunoblot analysis, lentivirus-mediated delivery of MEK1 shRNAs resulted in complete silencing of MEK1 expression with no effect on MEK2, whereas the two MEK2 shRNAs markedly knocked-down MEK2 expression without affecting MEK1 isoform (Fig. 6B). We then analyzed the functional consequence of MEK1 or MEK2 silencing on the proliferation rate of the cells. Strikingly, lowering of MEK2 expression with the two shRNAs completely suppressed the proliferation of HCT116 cells, whereas MEK1 shRNAs exerted a significant but much weaker effect (Fig. 6B). The extent of inhibition observed with MEK2 shRNAs was similar to that obtained by treating cells with the non-selective MEK1/2 inhibitor U0126. We also showed that silencing of MEK1 or MEK2 expression significantly reduces the extent of ERK1 and ERK2 activating phosphorylation (Fig. 7).

To verify whether this differential contribution of MEK isoforms could be generalized to other colorectal cancer cells, we examined the impact of MEK1 or MEK2 silencing on the proliferation of two other human colon cancer cell lines. We specifically chose the human colon carcinoma cell lines SW480 (which harbors a *KRAS *mutation like HCT116) and HT-29 (harboring a *BRAF *mutation). SW480 cells display a comparable expression pattern of MEK1 and MEK2 proteins as HCT116 cells (Fig. 6A). Similar to HCT116 cells, knock-down of MEK2 expression dramatically suppressed the proliferation of SW480 cells, whereas MEK1 silencing induced a significant but much lower decrease of cell proliferation (Fig. 6C). Similar results were obtained in HT-29 cells, except that the inhibitory effect of MEK1 shRNAs on proliferation was quantitatively more important than on HCT116 and SW480 cells (Fig. 6D). This observation could be explained by the much higher expression of MEK1 in the HT-29 cell line as compared to HCT116 or SW480 cells (Fig. 6A), which may have a more important contribution to total MEK1/2 signaling. However, the single inactivation of MEK2 was still capable of abolishing the proliferation of HT-29 cells even in the presence of high MEK1 levels. For all colorectal cancer cell lines tested, the inhibition of proliferation seen with MEK2 shRNAs was comparable to that achieved with the MEK1/2 inhibitor U0126.

To further extend our investigation to non-colorectal carcinomas, we tested the effect of MEK1 and MEK2 shRNAs on the human breast adenocarcinoma cell line MDA-MB-231, which exhibit strong constitutive activation of MEK1/MEK2 signaling (Fig. 6A). Interestingly, the MEK2 shRNA-06 completely inhibited the proliferation of MDA-MB-231 cells to the same extent as the drug inhibitor U0126 (Additional file 4). The other MEK2 shRNA-08 also markedly but not completely inhibited cell proliferation, consistent with its lower silencing activity in these cells. Expression of MEK1 shRNAs suppressed cell proliferation by approximately 50%.

## Discussion

The ERK1/2 MAP kinase signaling pathway plays a central role in cell proliferation control. Activation of ERK1/ERK2 is essential for G1 to S phase progression and is associated with induction of cyclin Ds and inhibition of anti-proliferative genes [51]. Studies in various experimental models have also implicated the Raf-MEK1/2-ERK1/2 pathway in the control of cell survival [52]. Consistent with a role in cell cycle and survival signaling, there is growing evidence that activation of the ERK1/2 pathway is involved in the pathogenesis of human cancer (see Introduction). Specifically, several observations point towards a role of this pathway in colorectal cancer [26]. First, the EGF receptor, a known activator of the ERK1/2 pathway, is expressed in more than 70% of colorectal cancers [53]; treatment with the EGF receptor monoclonal antibody cetuximab improves overall survival in patients with colorectal cancer [54]. Second, *KRAS *and *BRAF *genes are mutated in approximately 50% of colorectal cancers [3]. Third, activating phosphorylation of ERK1/ERK2 MAP kinases is frequently observed in human colorectal cancer cell lines and tumor specimens [20]. Finally, treatment with synthetic MEK1/2 inhibitors markedly attenuates the proliferation of colon carcinoma cells *in vitro *and in mouse xenografts [20]. Despite such evidence, several important questions about the contribution of the ERK1/2 MAP kinase pathway to the initiation and progression of colorectal cancer remain unanswered.

In this study, we show that constitutive activation of MEK1 or MEK2 isoform, as observed in 44% of colorectal cancers, is sufficient to fully transform normal intestinal epithelial cells and that maintenance of MEK1/MEK2 activity is necessary to sustain the proliferation of human colon carcinoma cells. This is the first report to compare the ability of the two MEK isoforms to transform epithelial cells. Previous studies have shown that activated MEK1 can transform immortalized fibroblasts [10,11] as well as epithelial cells [13,55,56]. Intriguingly, it was also reported that activated Ras, but not Raf-1, causes transformation of mammary and intestinal epithelial cells, suggesting that signaling events other than activation of MEK1/2 are essential for oncogenic Ras transformation [57]. Here, we clearly establish that expression of activated MEK1 is sufficient to morphologically transform intestinal epithelial cells, accelerate cell proliferation, and induce the rapid formation of aggressive tumors after orthotopic transplantation. Moreover, we reveal for the first time that the MEK2 isoform has similar transforming properties and is able to induce the formation of tumors in mice. This knowledge is important since both MEK1 and MEK2 are expressed in intestinal epithelial cells and immunohistochemistry analysis with phospho-specific MEK1/2 antibodies does not allow to discriminate between the two isoforms. Immunoblot analysis under electrophoresis conditions that partially resolve the two isoforms indicates that both MEK1 and MEK2 are phosphorylated in human colon carcinoma cell lines (Fig. 6A).

The signaling pathways underlying the progression of colorectal cancer to advanced metastatic disease are poorly understood. The development of metastatic tumors is a complex process that consists of a series of cellular events that move neoplastic cells from the primary tumor to a distant location [58]. Cancer cells must detach from the tumor and invade the surrounding tissue, degrade the basement membrane, disseminate and survive into the circulation systems, extravasate into a new tissue, and colonize their new microenvironment. During this process, tumor cells have to face different kinds of stress. Recent studies have suggested that Ras signaling may contribute to metastasis formation in colorectal cancer [59], although the downstream effector pathways involved remain unclear. Here, we show that expression of activated MEK1 or MEK2 not only induces the formation of intestinal tumors but also promotes later stages of tumor progression and metastasis to distant organs. To address the impact of MEK1/MEK2 signaling on tumor progression, we have used an orthotopic implantation model that provides a more accurate picture of the metastatic process [60]. A large proportion of the tumors expressing activated MEK1 or MEK2 metastasized to the liver and lung, the two most common sites of human colorectal cancer metastasis, thereby validating the pathological relevance of our model. The ability of activated MEK1- or MEK2-expressing tumor cells to colonize distant organs was associated with increased invasiveness, secretion of matrix proteases and resistance to anoikis. Interestingly, an early study reported that the enzymatic activity of ERK1/ERK2 is markedly up-regulated during late progression of carcinogen-induced colon carcinomas [61]. Together, these observations strengthen the idea that ERK1/2 MAP kinase signaling plays a critical role in colorectal cancer progression [26].

An important finding of this study is the observation that MEK1 and MEK2 may contribute differentially to the pathogenesis of colorectal cancer. While activation of a single MEK isoform was shown to be sufficient for full neoplastic transformation of intestinal epithelial cells, we observed that MEK2DD-expressing cells display a slightly more transformed morphology and are more invasive than cells expressing MEK1DD *in vitro*. This was associated with a more prominent down-regulation of E-cadherin and a stronger up-regulation of MMPs and urokinase receptor. The physiopathological relevance of these *in vitro *properties is unclear, however, since no difference in the metastatic behavior of the cells was observed upon orthotopic transplantation in mice. The simplest explanation for this apparent discrepancy is that *in vitro *invasiveness assays do not replicate the complex and multistage process of tumor metastasis *in vivo*. Most importantly, we found that silencing of MEK2 expression (even if not total) completely suppressed the proliferation of human colon carcinoma cell lines, whereas complete knock-down of MEK1 had a much weaker effect. The extent of inhibition observed with MEK2 shRNAs was comparable to that obtained with the non-selective MEK1/2 inhibitor U0126. This differential impact of MEK1 and MEK2 on cell proliferation was observed in three different colon carcinoma cell lines (bearing activating mutations in either *KRAS *or *BRAF*), as well as in the breast adenocarcinoma cell line MDA-MB-231. The molecular basis for these differences remains to be established. One possibility is that MEK2 is expressed at higher levels than MEK1 in colon cancer cells. However, immunoblot analysis clearly indicates that HT-29 cells express more phosphorylated MEK1 (lower band in upper panel of Fig. 6A) than MEK2, arguing that quantitative difference in expression levels does not explain everything. Our results rather suggest that the two MEK isoforms may be differentially regulated or may target distinct effector pathways in certain cellular and/or genetic contexts.

## Conclusion

In conclusion, we demonstrate that the two MAP kinase kinase isoforms MEK1 and MEK2 have similar transforming properties and that activation of either isoform is sufficient for full transformation of intestinal epithelial cells up to the metastatic stage. Interestingly, our results indicate that MEK2 plays a more important role than MEK1 in sustaining the proliferation of human colorectal cancer cells, suggesting that the two MEK isoforms may contribute differentially to tumor pathogenesis in certain contexts.

## Competing interests

The authors declare that they have no competing interests.

## Authors' contributions

LV participated to most of the experiments and contributed to study design, analysis of the data and drafting of the manuscript. CJ and SD carried out most of the RNA interference experiments and participated in the cellular and biochemical assays. KG and IC conducted cellular and biochemical assays. MKSL contributed to the RNA interference experiments and animal studies. IGRG and DL contributed to the RNA interference experiments. LG carried out the histopathological analysis of intestinal tumors. MB carried out the immunohistochemistry analysis and contributed to study conception. SM conceived the study, was responsible for its coordination and for preparation of the final manuscript. All authors read and approved the manuscript.

## Pre-publication history

The pre-publication history for this paper can be accessed here:



## Supplementary Material

Additional File 1 **List of up-regulated and down-regulated genes in IEC-6 cells expressing MEK1DD.** Click here for file

Additional File 2 **List of up-regulated and down-regulated genes in IEC-6 cells expressing MEK2DD.** Click here for file

Additional File 3 **HCT116 cells were infected with lentiviruses encoding shRNAs to MEK1 or MEK2 gene.** Expression of MEK isoforms was analyzed by immunoblotting 5 days after infection. Click here for file

Additional File 4 **Impact of MEK1 or MEK2 silencing on the proliferation of a breast carcinoma cell line.** MDA-MB-231 cells were infected with MEK1 or MEK2 shRNA-encoding lentiviruses or treated with the MEK1/2 inhibitor U0126. Expression of MEK isoforms and cell proliferation were measured as described in the legend of Figure 6. Click here for file
