# Supplementary material for: Activation of MEK1 or MEK2 isoform is sufficient to fully transform intestinal epithelial cells and induce the formation of metastatic tumors
Source: BMC Cancer. 2008 Nov 17;8:337. doi: 10.1186/1471-2407-8-337 (PMC2596176; doi:10.1186/1471-2407-8-337)
Supplement: Additional File 1 — List of up-regulated and down-regulated genes in IEC-6 cells expressing MEK1DD. [file 1471-2407-8-337-S1.pdf]

Additional File 1

List of up-regulated and down-regulated genes in IEC-6 cells expressing MEK1DD.

Gene symbol	Gene name	Fold change	*P*-value	Function
Aldh3a1	aldehyde dehydrogenase family 3, member A1	17,01	<0,001	aldehyde dehydrogenase activity
LOC494499	LOC494499 protein	11,48	<0,001	NA
Cyp3a9	cytochrome P450, family 3, subfamily a, polypeptide 9	10,06	<0,001	monooxygenase activity
Serpine2	serine (or cysteine) proteinase inhibitor, clade E, member 2	9,12	<0,001	serine-type endopeptidase inhibitor activity
Aqp1	aquaporin 1	8,25	<0,001	transporter activity
Spp1	secreted phosphoprotein 1	7,14	<0,001	cytokine activity
Pla2g2a	phospholipase A2, group IIA (platelets, synovial fluid)	6,65	<0,001	phospholipase A2 activity
Mmp13	matrix metallopeptidase 13	5,56	<0,001	metalloendopeptidase activity
P2ry2	purinergic receptor P2Y, G-protein coupled 2	4,75	<0,001	rhodopsin-like receptor activity
LOC290595	hypothetical gene supported by AF152002	4,30	<0,001	NA
Gsta4	glutathione S-transferase, alpha 4	4,21	<0,001	glutathione transferase activity
Cpt1a	carnitine palmitoyltransferase 1a, liver	3,78	<0,001	carnitine O-palmitoyltransferase activity
Lgals4	lectin, galactose binding, soluble 4	3,78	<0,001	sugar binding
St6gal1	beta galactoside alpha 2,6 sialyltransferase 1	3,61	<0,001	sialyltransferase activity
Mmp3	matrix metallopeptidase 3	3,57	<0,001	metalloendopeptidase activity
Ca2	carbonic anhydrase 2	3,55	<0,001	carbonate dehydratase activity
Cd9	CD9 antigen	3,54	<0,001	protein binding
Agc1	aggrecan 1	3,45	<0,001	extracellular matrix structural constituent
Hmga1	high mobility group AT-hook 1	3,41	<0,001	NA
RGD1311155	similar to RIKEN cDNA 9230117N10	3,37	<0,001	NA
Pthlh	parathyroid hormone-like peptide	3,35	<0,001	hormone activity
Fosl1	fos-like antigen 1	3,30	<0,001	DNA binding
Adh1	alcohol dehydrogenase 1 (class I)	3,27	<0,001	alcohol dehydrogenase activity
Scd1	stearoyl-Coenzyme A desaturase 1	3,14	<0,001	stearoyl-CoA 9-desaturase activity
Gchfr	GTP cyclohydrolase I feedback regulator	3,07	<0,001	enzyme inhibitor activity
LOC682651	similar to Metallothionein-2 (MT-2)	3,06	<0,001	NA
Ctsl	cathepsin L	2,94	<0,001	cysteine-type endopeptidase activity
Foxq1	forkhead box Q1	2,92	<0,001	DNA binding
Pparg	peroxisome proliferator activated receptor gamma	2,83	<0,001	DNA binding
Adfp	Adipose differentiation related protein	2,81	<0,001	long-chain fatty acid transport
Tgfa	transforming growth factor alpha	2,75	<0,001	epidermal growth factor receptor binding
Aplp2	amyloid beta (A4) precursor-like protein 2	2,69	<0,001	DNA binding
Krt2-8	keratin complex 2, basic, gene 8	2,67	<0,001	protein kinase activity
Serpinb2	serine (or cysteine) proteinase inhibitor, clade B, member 2	2,65	<0,001	serine-type endopeptidase inhibitor activity
Pfkp	phosphofructokinase, platelet	2,54	<0,001	6-phosphofructokinase activity
Igfbp2	insulin-like growth factor binding protein 2	-11,20	<0,001	insulin-like growth factor binding
Tagln	transgelin	-9,92	<0,001	protein binding, bridging
Fbln5	fibulin 5	-7,76	<0,001	calcium ion binding
Edn1	endothelin 1	-6,75	<0,001	protein binding
Acta2	smooth muscle alpha-actin	-6,28	<0,001	protein binding
Ankrd1	ankyrin repeat domain 1 (cardiac muscle)	-5,80	<0,001	NA
Dcn	decorin	-5,00	<0,001	collagen binding
Plagl1	pleiomorphic adenoma gene-like 1	-4,43	<0,001	nucleic acid binding
Ddah1	dimethylarginine dimethylaminohydrolase 1	-4,37	<0,001	zinc ion binding
Bmp6	bone morphogenetic protein 6	-3,97	<0,001	cytokine activity
Hapln1	hyaluronan and proteoglycan link protein 1	-3,76	<0,001	hyaluronic acid binding
Col1a2	Procollagen, type I, alpha 2	-3,17	<0,001	structural molecule activity
LOC313672	similar to CG11206-PA	-2,98	<0,001	NA
Dusp1	dual specificity phosphatase 1	-2,94	<0,001	phosphoprotein phosphatase activity
Col12a1	procollagen, type XII, alpha 1	-2,87	<0,001	structural molecule activity
Vsnl1	visinin-like 1	-2,77	<0,001	calcium ion binding
Pak1	p21 (CDKN1A)-activated kinase 1	-2,70	<0,001	protein kinase activity
Cd200	Cd200 antigen	-2,70	<0,001	protein binding
Col3a1	procollagen, type III, alpha 1	-2,64	<0,001	structural molecule activity
Ptgis	prostaglandin I2 (prostacyclin) synthase	-2,62	<0,001	monooxygenase activity
Anxa3	annexin A3	-2,57	<0,001	phospholipase inhibitor activity
