# Supplementary material for: Activation of MEK1 or MEK2 isoform is sufficient to fully transform intestinal epithelial cells and induce the formation of metastatic tumors
Source: BMC Cancer. 2008 Nov 17;8:337. doi: 10.1186/1471-2407-8-337 (PMC2596176; doi:10.1186/1471-2407-8-337)
Supplement: Additional File 2 — List of up-regulated and down-regulated genes in IEC-6 cells expressing MEK2DD. [file 1471-2407-8-337-S2.pdf]

Additional File 2

List of up-regulated and down-regulated genes in IEC-6 cells expressing MEK2DD.

Gene symbol	Gene name	Fold change	*P*-value	Function
Mmp13	matrix metallopeptidase 13	14,33	<0,001	metalloendopeptidase activity
Gchfr	GTP cyclohydrolase I feedback regulator	9,81	<0,001	enzyme inhibitor activity
Serpinb2	serine (or cysteine) proteinase inhibitor, clade B, member 2	9,00	<0,001	serine-type endopeptidase inhibitor activity
Spp1	secreted phosphoprotein 1	8,94	<0,001	cytokine activity
Mmp3	matrix metallopeptidase 3	8,46	<0,001	metalloendopeptidase activity
Serpine2	serine (or cysteine) proteinase inhibitor, clade E, member 2	8,19	<0,001	serine-type endopeptidase inhibitor activity
Aldh3a1	aldehyde dehydrogenase family 3, member A1	7,79	<0,001	aldehyde dehydrogenase activity
Mmp10	matrix metallopeptidase 10	7,76	<0,001	metalloendopeptidase activity
Aqp1	aquaporin 1	7,51	<0,001	transporter activity
RGD1311155	similar to RIKEN cDNA 9230117N10	5,88	<0,001	NA
Tgfa	transforming growth factor alpha	5,76	<0,001	epidermal growth factor receptor binding
Agc1	aggrecan 1	5,33	<0,001	extracellular matrix structural constituent
Prss22_predicted	protease, serine, 22 (predicted)	5,28	<0,001	NA
Fosl1	fos-like antigen 1	5,07	<0,001	DNA binding
Hmga1	high mobility group AT-hook 1	4,83	<0,001	NA
Areg	amphiregulin	4,75	<0,001	cytokine activity
Ca2	carbonic anhydrase 2	4,16	<0,001	carbonate dehydratase activity
Fst	follistatin	4,12	<0,001	activin binding
Cpe	carboxypeptidase E	4,07	<0,001	carboxypeptidase activity
Ercc1_predicted	excision repair cross-complementing rodent repair deficiency	4,01	<0,001	NA
Rasa1	RAS p21 protein activator 1	3,95	<0,001	GTPase activator activity
Cyp3a9	cytochrome P450, family 3, subfamily a, polypeptide 9	3,91	<0,001	monooxygenase activity
Gsta4	glutathione S-transferase, alpha 4	3,79	<0,001	glutathione transferase activity
Btg1	B-cell translocation gene 1, anti-proliferative	3,73	<0,001	transcription cofactor activity
St6gal1	beta galactoside alpha 2,6 sialyltransferase 1	3,68	<0,001	sialyltransferase activity
Adfp	Adipose differentiation related protein	3,62	<0,001	NA
Cpt1a	carnitine palmitoyltransferase 1a, liver	3,62	<0,001	carnitine O-palmitoyltransferase activity
LOC494499	LOC494499 protein	3,56	<0,001	NA
Ptprn	protein tyrosine phosphatase, receptor type, N	3,34	<0,001	phosphoprotein phosphatase activity
Cd24	CD24 antigen	3,31	<0,001	NA
Plcd4	phospholipase C, delta 4	3,22	<0,001	phosphoinositide phospholipase C activity
Mgp	matrix Gla protein	3,13	<0,001	calcium ion binding
Nrn1	neuritin	3,07	<0,001	NA
Cd82	CD82 antigen	3,06	<0,001	NA
Cd9	CD9 antigen	3,06	<0,001	protein binding
Lnk	linker of T-cell receptor pathways	2,95	<0,001	protein binding
Ccr1	chemokine (C-C motif) receptor 1	2,94	<0,001	rhodopsin-like receptor activity
RGD1562618_predicted	similar to RIKEN cDNA 6030419C18 gene (predicted)	2,84	<0,001	NA
Fcgr3	Fc receptor, IgG, low affinity III	2,77	<0,001	receptor activity
Sc4mol	sterol-C4-methyl oxidase-like	2,75	<0,001	C-4 methylsterol oxidase activity
Sod3	superoxide dismutase 3, extracellular	2,72	<0,001	superoxide dismutase activity
S100a4	S100 calcium-binding protein A4	2,69	<0,001	calcium ion binding
Ctsl	cathepsin L	2,65	<0,001	cysteine-type endopeptidase activity
Pter	phosphotriesterase related	2,64	<0,001	aryldialkylphosphatase activity
Sh3kbp1	SH3-domain kinase binding protein 1	2,62	<0,001	protein binding
Scd1	stearoyl-Coenzyme A desaturase 1	2,61	<0,001	stearoyl-CoA 9-desaturase activity
Fkhl18	forkhead-like 18 (Drosophila)	2,57	<0,001	transcription factor activity
Plaur	plasminogen activator, urokinase receptor	2,53	<0,001	receptor activity
Tagln	transgelin	-13,40	<0,001	protein binding, bridging
Igfbp2	insulin-like growth factor binding protein 2	-11,83	<0,001	insulin-like growth factor binding
Fbln5	fibulin 5	-11,71	<0,001	calcium ion binding
Edn1	endothelin 1	-10,70	<0,001	protein binding
Ankrd1	ankyrin repeat domain 1 (cardiac muscle)	-9,01	<0,001	NA
Acta2	smooth muscle alpha-actin	-8,52	<0,001	protein binding
Dcn	decorin	-6,44	<0,001	collagen binding
Cryab	crystallin, alpha B	-5,36	<0,001	structural constituent of eye lens
Metrnl	meteorin, glial cell differentiation regulator-like	-5,28	<0,001	NA
Bmp6	bone morphogenetic protein 6	-4,24	<0,001	cytokine activity
Dusp1	dual specificity phosphatase 1	-4,18	<0,001	phosphoprotein phosphatase activity
Hapln1	hyaluronan and proteoglycan link protein 1	-4,06	<0,001	hyaluronic acid binding
Ddah1	dimethylarginine dimethylaminohydrolase 1	-4,02	<0,001	zinc ion binding
Clu	clusterin	-4,02	<0,001	NA
Col12a1	procollagen, type XII, alpha 1	-4,00	<0,001	structural molecule activity
Prnp	prion protein	-3,99	<0,001	copper ion binding
Bmp4	bone morphogenetic protein 4	-3,94	<0,001	cytokine activity
LOC313672	similar to CG11206-PA	-3,91	<0,001	NA
Man1a_predicted	mannosidase 1, alpha (predicted)	-3,84	<0,001	calcium ion binding
Rhob	ras homolog gene family, member B	-3,43	<0,001	GTP binding
Vsnl1	visinin-like 1	-3,22	<0,001	calcium ion binding
Cald1	caldesmon 1	-3,21	<0,001	actin binding
Cmkor1	chemokine orphan receptor 1	-3,19	<0,001	rhodopsin-like receptor activity
Pawr	PRKC, apoptosis, WT1, regulator	-3,16	<0,001	protein binding
Vldlr	very low density lipoprotein receptor	-3,13	<0,001	receptor activity
Grb14	growth factor receptor bound protein 14	-3,10	<0,001	SH3/SH2 adaptor activity
Myl9_predicted	myosin, light polypeptide 9, regulatory (predicted)	-3,05	<0,001	calcium ion binding
Pak1	p21 (CDKN1A)-activated kinase 1	-2,99	<0,001	protein kinase activity
Loxl1	lysyl oxidase-like 1	-2,98	<0,001	protein-lysine 6-oxidase activity
Anxa3	annexin A3	-2,95	<0,001	phospholipase inhibitor activity
App	amyloid beta (A4) precursor protein	-2,91	<0,001	serine-type endopeptidase inhibitor activity
Rgc32	response gene to complement 32	-2,81	<0,001	protein binding
Sparc	secreted acidic cysteine rich glycoprotein	-2,80	<0,001	calcium ion binding
Plagl1	pleiomorphic adenoma gene-like 1	-2,77	<0,001	nucleic acid binding
Lox	lysyl oxidase	-2,73	<0,001	protein-lysine 6-oxidase activity
Tmeff1	transmembrane protein with EGF-like and two follistatin-like domains 1	-2,73	<0,001	serine-type endopeptidase inhibitor activity
Fah	fumarylacetoacetate hydrolase	-2,70	<0,001	DNA binding
Rnd3	Rho family GTPase 3	-2,70	<0,001	GTP binding
Cd200	Cd200 antigen	-2,60	<0,001	protein binding
Ptgis	prostaglandin I2 (prostacyclin) synthase	-2,57	<0,001	monooxygenase activity
Cdc42ep3_predicted	CDC42 effector protein (Rho GTPase binding) 3 (predicted)	-2,53	<0,001	NA
Fyn	fyn proto-oncogene	-2,53	<0,001	protein kinase activity
Pdpn	podoplanin	-2,51	<0,001	water transporter activity
