# Supplementary figures and images for: Activation of MEK1 or MEK2 isoform is sufficient to fully transform intestinal epithelial cells and induce the formation of metastatic tumors
Source: BMC Cancer. 2008 Nov 17;8:337. doi: 10.1186/1471-2407-8-337 (PMC2596176; doi:10.1186/1471-2407-8-337)

## Additional File 3

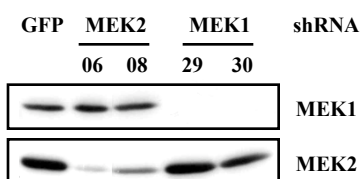

Supplement: Additional File 3 — HCT116 cells were infected with lentiviruses encoding shRNAs to MEK1 or MEK2 gene. Expression of MEK isoforms was analyzed by immunoblotting 5 days after infection. [file 1471-2407-8-337-S3.pdf]

## Additional File 4

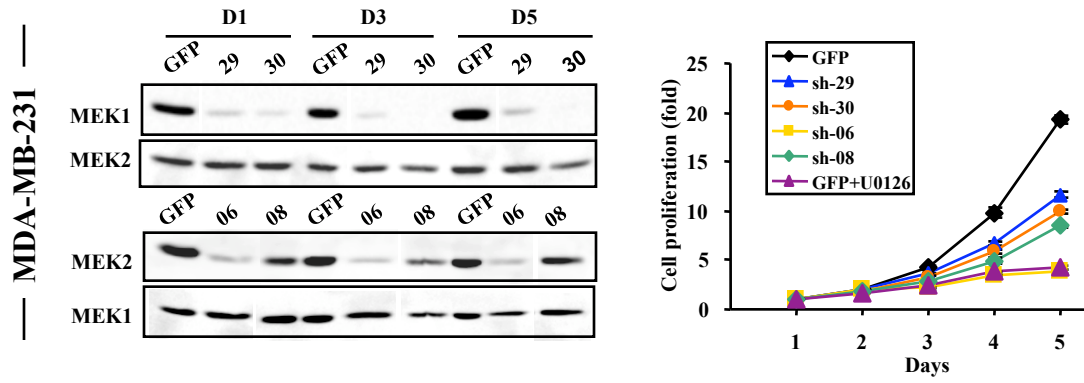

Supplement: Additional File 4 — Impact of MEK1 or MEK2 silencing on the proliferation of a breast carcinoma cell line. MDA-MB-231 cells were infected with MEK1 or MEK2 shRNA-encoding lentiviruses or treated with the MEK1/2 inhibitor U0126. Expression of MEK isoforms and cell proliferation were measured as described in the legend of Figure 6. [file 1471-2407-8-337-S4.pdf]
